# The association between dyslipidemia and the incidence of chronic kidney disease in the general Zhejiang population: a retrospective study

**DOI:** 10.1186/s12882-020-01907-5

**Published:** 2020-07-02

**Authors:** Xudong Liang, Meiyu Ye, Mei Tao, Danna Zheng, Ruyi Cai, Yifan Zhu, Juan Jin, Qiang He

**Affiliations:** 1 Department of Nephrology, Zhejiang Provincial People’s Hospital, No.158th, Shangtang Road, Xiacheng, Hangzhou, Zhejiang 310014 P.R. China; 2 People’s Hospital of Hangzhou Medical College, Hangzhou, Zhejiang 310014 P.R. China; 3 Chinese Medical Nephrology Key Laboratory of Zhejiang Province, Hangzhou, Zhejiang 310014 P.R. China; 4 Department of Nephrology, The Second Hosipital of Yinzhou, Ningbo, Zhejiang 315192 P.R. China

**Keywords:** Renal function, Lipid profiles, Epidemiology, eGFR

## Abstract

**Background:**

According to the “lipid nephrotoxicity hypothesis”, there is now significant research being conducted in this area. By studying the role of hyperlipidemia in chronic kidney disease in the general Zhejiang population, we aimed to explore the correlation between changes in blood lipid levels and chronic kidney disease.

**Methods:**

We collected and analyzed clinical data from ordinary residents who participated in the annual comprehensive physical examination with no overt kidney disease in Zhejiang Provincial People’s Hospital, China from January 2011 to December 2016. According to triglyceride, total cholesterol and low-density lipoprotein levels, participants were respectively divided into 4 groups. Statistical methods were used to evaluate the correlation between different blood lipid profiles and chronic kidney disease.

**Results:**

Five thousand one hundred eighty-three participants were included in our study. During the six-year follow-up period, 227 participants (4.4%) developed chronic kidney disease. The odds ratio for incident chronic kidney disease was 3.14 (95%CI: 1.53–6.43) in Q3, 3.84 (95%CI: 1.90–7.76) in Q4 according to the total cholesterol group and 1.17 (95%CI: 1.04–1.32) in Q3, 1.40 (95%CI: 1.11–2.48) in Q4 according to the low-density lipoprotein group, respectively, after multivariable-adjusted analyses. According to the triglyceride grouping, the odds ratio for incident chronic kidney disease was 2.88 (95%CI: 1.29–6.43) in Q2, 2.92 (95%CI: 1.44–6.57) in Q3 and 3.08 (95%CI: 1.11–6.69) in Q4, after multivariable-adjusted analyses.

**Conclusion:**

Increased triglycerides and high levels of total cholesterol and low-density lipoprotein were independently associated with an increased likelihood of estimated glomerular filtration rate (eGFR) decline and development of incident chronic kidney disease in the general Zhejiang population.

## Background

The prevalence of chronic kidney disease (CKD) in China and the rest of the world is increasing [1]. It has been estimated that the prevalence of CKD in China is approximately 10.8% [2]. CKD not only causes end-stage renal disease (ESRD), which requires renal replacement therapy, but also induces complications and increases mortality [3]. Therefore, risk factors for CKD should be detected early, managed and controlled, which can significantly prevent the development of CKD [4].

Due to the continuous changes in living standards and dietary habits, the incidence of dyslipidemia has increased annually. Dyslipidemia includes hypertriglyceridemia, hypercholesterolemia, lower high-density lipoproteinemia, and mixed hyperlipidemia [5]. Many studies have shown that dyslipidemia can lead to cardiovascular disease (CVD) [6, 7].

When the “Lipid Nephrotoxicity Hypothesis” was proposed in 1982, CKD was postulated as being associated with dyslipidemia [8]. Over the last few decades, this area has been continuously developed and researched [9, 10]. Large-scale studied in the 1990s showed that proteinuria increased in CKD patients with dyslipidemia after 10 years of follow-up [11]. A study had evaluated dietary patterns and CKD risk. After more than 6 years of follow-up, high-fat and high-sugar diets will increase the incidence of CKD (46%), while vegetarian diets can reduce the incidence of CKD (43%) [12]. Similarly, a study showed that a diet based on fat and sweets was positively correlated with decreased renal function [13].

However, there have been conflicting results from different studies. Whether triglycerides (TG), total cholesterol (TC) and low-density lipoprotein (LDL) have any influence on the development of CKD is still unclear, and to what extent. It has not been established whether elevated blood lipid levels are an independent risk factor for incident CKD or whether a rapid decline in annual estimated glomerular filtration rate (eGFR) in non-CKD individuals results in CKD. Furthermore, little is known on which types of blood lipids have the greatest impact on incident CKD [14–16].

In this study, the retrospective associations of serum lipid levels with progression of renal dysfunction and incident CKD were investigated in a large cohort based on annual physical examination of participants with eGFR ≥60 mL/min/1.73 m^2^ and no basal kidney disease. In addition, we also performed an analysis of the different groupings of blood lipid types.

## Methods

The study design is a retrospective observational study based on the general Chinese population. Participants were 25–85 years old, worked in Hangzhou City and participated in a comprehensive annual medical examination at the Zhejiang Provincial People’s Hospital, China from January 2011 to December 2016. The baseline eGFR was ≥60 ml/min/1.73m^2^.

### Study population

**Fig. 1:**
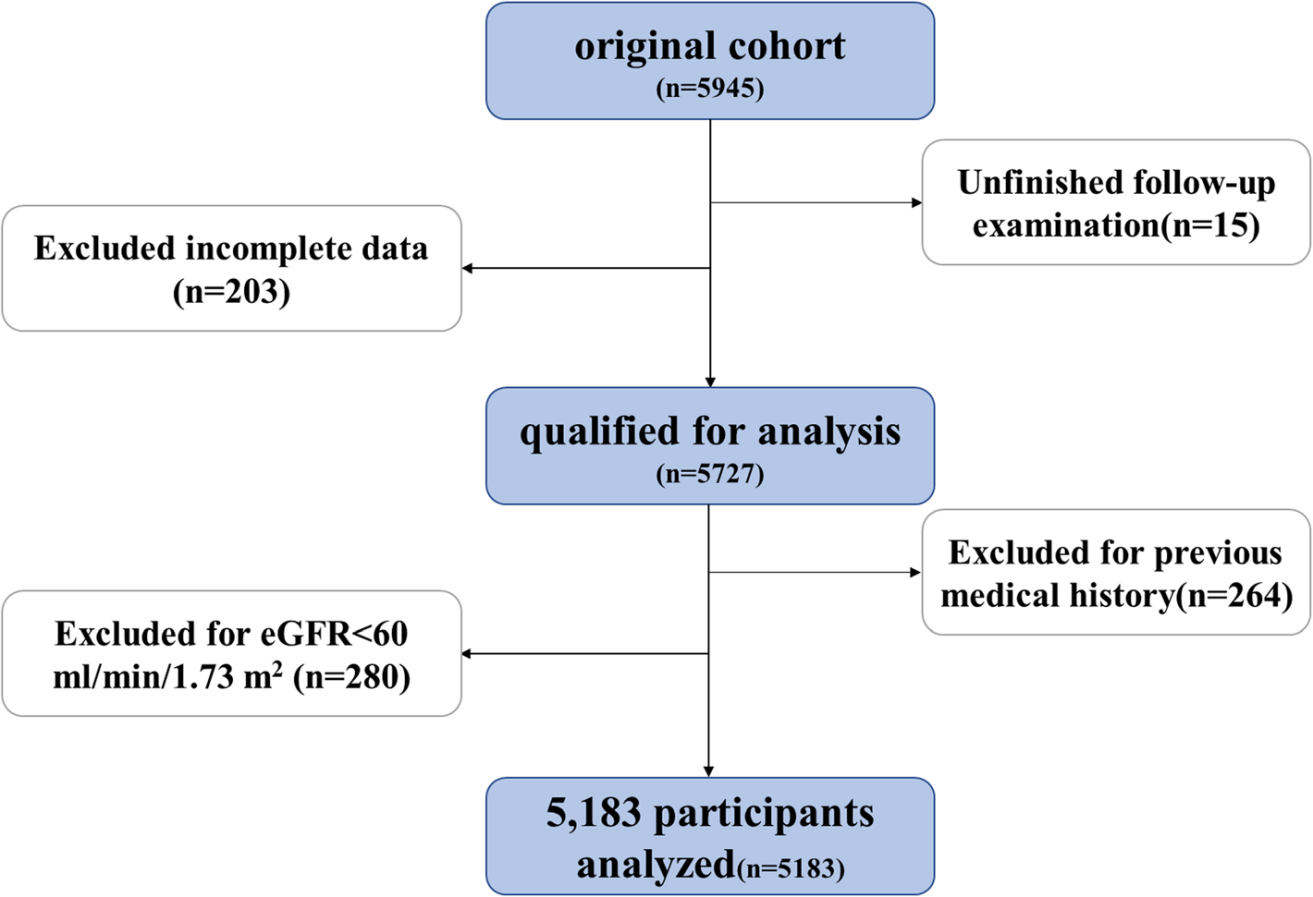
Flow diagram of study population

A total of 5945 participants had complete baseline data. However, 15 of these failed to complete the follow-up examination. Then, we deleted 203 participants with incomplete data. In addition, 264 patients were excluded for previous medical history including: cardiovascular disease, cerebrovascular disease, severe liver damage, severe infection, malignancy, kidney surgery or other life-threatening illnesses. For the remaining 5463 participants, 280 individuals were excluded due to baseline eGFR < 60 ml/min/1.73 m^2^. Finally, a total of 5183 participants were selected for the research analysis (Fig. 1).

### Data collection and analysis

Baseline characteristics of participants included age, gender, biochemical measurements, and medical history. Weight and height were measured while the participants were wearing lightweight clothes with no shoes. The body mass index (BMI) was calculated as the weight (kg) divided by the square of the height (m). After 15 min of rest, the participants were placed in a sitting position, and systolic and diastolic blood pressure (SBP/DBP) were measured using an automatic sphygmomanometer. Blood pressure (BP) was measured twice, with an interval of more than 5 min, then the mean value was selected. Blood samples were taken after over 12 h of fasting overnight. We measured biomedical parameters using a biochemical automatic enzyme analyzer, including blood urea nitrogen (BUN), fasting blood glucose (FPG), TC, TG, high-density lipoprotein (HDL), LDL, and uric acid (SUA) in the Clinical Laboratory of Zhejiang Provincial People’s Hospital. All covariates were measured once at baseline. All indicators were examined during follow-up. Renal function was calculated by using the formula for eGFR (ml / min / 1.73 m^2^) = 186 × SCr ^- 1.154^ × age ^- 0.203^ (× 0.742, if female), which is derived from the simplified Modification of Diet in Renal Disease (MDRD) study for Chinese people [17]. According to the Kidney Disease: Improving Global Outcomes (KDIGO) 2012 Clinical Practice Guideline, an incident CKD event was defined as eGFR < 60 ml / min / 1.73 m^2^ during follow-up. For individuals who had more than one CKD event during the follow-up period, only the first event was included in our statistical analysis.

### Statistical analysis

All statistical analyses were performed using SPSS, version 23.0 (IBM, USA). All *P*-values were two-tailed, and *P* < 0.05 was considered to indicate statistical significance. Multivariate linear regression analysis was used to evaluate the association between blood lipid levels and eGFR changes. Baseline characteristics with normal distribution were reported as the mean (±standard deviation, SD) and percentage. Non-normally distributed variables were presented as the median with interquartile range (IQR). Independent-samples T-test was used to compare those with and without new incidence of CKD. The statistical differences between the baseline characteristics in relation to the quartiles of blood lipids levels was analyzed by using One-way ANOVA test for continuous variables, and Chi-square test for discrete variables. Multivariate linear regression analysis was used to evaluate the association between blood lipids levels and eGFR change. Multivariable-adjusted logistic regression analyses were employed to estimate odds ratio (*OR*) and 95% confidence interval (95% CI) for the new-onset CKD.

## Results

### Baseline characteristics

**Fig. 2:**
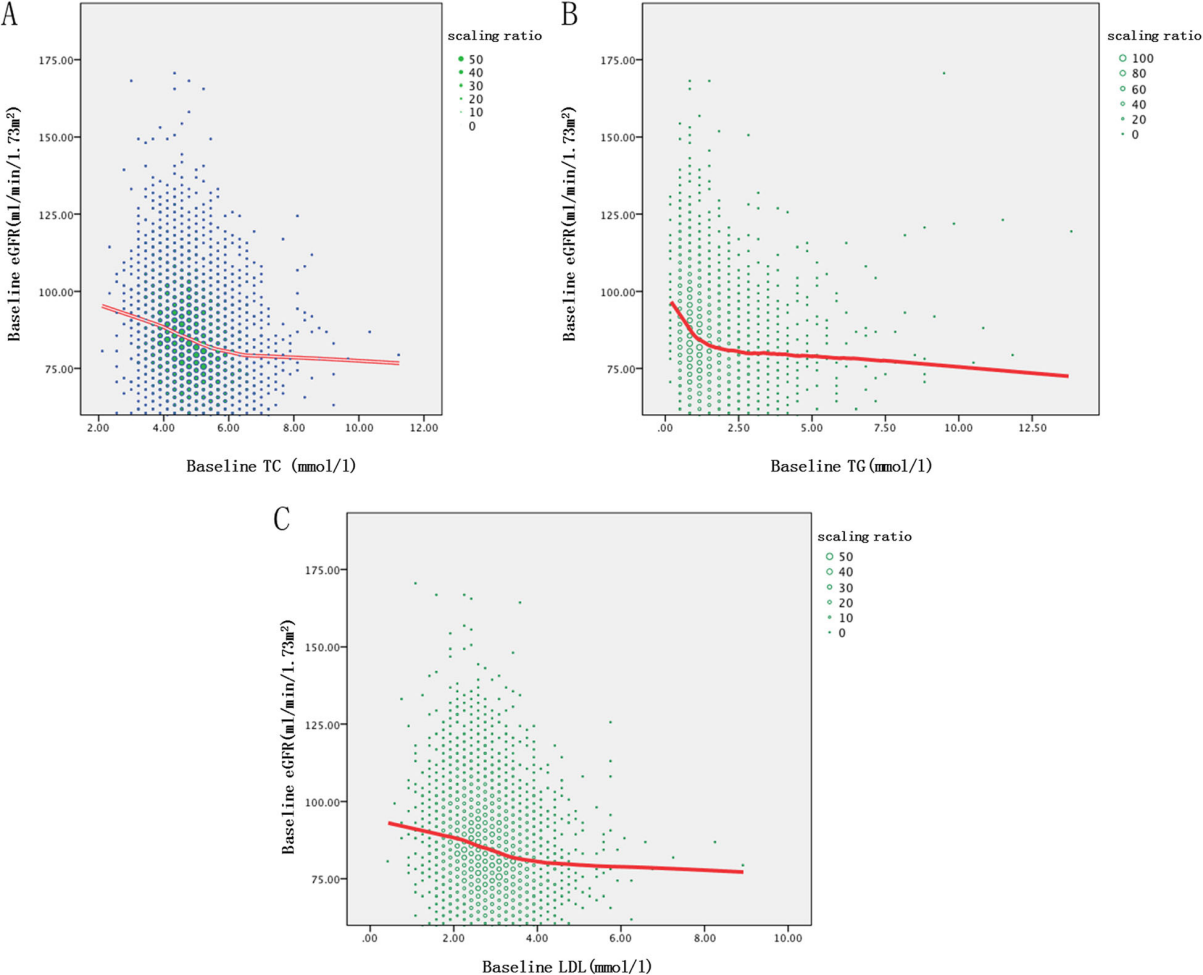
The relationship between blood lipid levels and eGFR at first visit. **a** The relationship between baseline TC and eGFR. **b** The relationship between baseline TG and eGFR. **c** The relationship between baseline LDL and eGFR

The distribution of the blood lipid levels at baseline is shown in Fig. 2. Baseline eGFR decreased with increasing baseline TC, TG, and LDL.

According to the age, it is divided into three groups: young people (≤ 40 years old), middle-aged (> 40, ≤ 60 years old), old people (> 60 years old) (Table 1).

**Table 1:** Comparison of relevant datas after age grouping

Age (Years)	Q1(≤40) (***n*** = 1862)	Q2(> 40, ≤ 60) (***n*** = 2135)	Q3(> 60) (***n*** = 1186)	***P*** value
Age (Years)	34(6)	51(9)^*^	72(14)^*#^	< 0.001
Male (%)	1209(64.9)	1265(59.3)^*^	702(59.2)^*^	< 0.001
BMI (kg/m^2^)	22.48 ± 3.05	23.59 ± 2.89^*^	23.81 ± 2.98^*#^	< 0.001
Overweight (24 ≤ BMI < 28) (%)	479(25.7)	754(35.3)^*^	498(42.0)^*#^	< 0.001
Obesity (BMI ≥ 28) (%)	76(4.1)	151(7.1)^*^	84(7.1)^*^	< 0.001
SBP (mmHg)	119.05 ± 14.21	122.67 ± 16.3^*^	140.61 ± 19.50^*#^	< 0.001
DBP (mmHg)	71.85 ± 9.59	76.10 ± 11.72^*^	80.37 ± 11.10^*#^	< 0.001
TG (mmol/L)	1.18 ± 0.91	1.57 ± 1.33^*^	1.61 ± 1.06^*#^	< 0.001
TC (mmol/L)	4.49 ± 0.85	4.93 ± 0.89^*^	5.16 ± 0.93^*#^	0.001
HDL-C (mmol/L)	1.30 ± 0.29	1.28 ± 0.31^*^	1.31 ± 0.32^#^	0.002
LDL-C (mmol/L)	2.58 ± 0.71	2.88 ± 0.74^*^	3.01 ± 0.78^*#^	< 0.001
BUN (mmol/L)	4.76 ± 1.06	4.99 ± 1.12^*^	5.38 ± 1.19^*#^	< 0.001
FPG (mmol/L)	4.94 ± 0.40	5.24 ± 0.83^*^	5.70 ± 1.08^*#^	< 0.001
SCr (μmol/L)	82.57 ± 13.80	82.53 ± 13.91	83.60 ± 13.20^*#^	0.068
SUA (mg/dl)	5.48 ± 1.32	5.58 ± 1.38^*^	5.74 ± 1.32^*#^	< 0.001
Hypertension (%)	10(0.5)	294(13.8)^*^	545(46.0)^*#^	< 0.001
Diabetes (%)	2(0.1)	58(2.7)^*^	138(11.6)^*#^	< 0.001
eGFR (ml/min/1.73m^2^)	93.87 ± 14.90	87.79 ± 14.00^*^	86.82 ± 11.77^*#^	< 0.001
New incident CKD (%)	43(2.3)	89(4.2)^*^	95(8.0)^*#^	< 0.001

^*^Compared with Q1 group, *P* < 0.05. ^#^Compared with Q2 group, *P* < 0.05

According to the age group, the prevalence of BMI, blood lipids, uric acid, urea nitrogen, blood sugar, diabetes, and hypertension in the youth group is lower than that in the middle-aged group and the elderly group, while eGFR is higher.

The baseline characteristics of the blood lipid groups are shown in Tables 2, 3 and 4. In general, the majority of participants were middle-aged males (30.8%). The average eGFR was 88.5 ml/min/1.73m^2^. Participants in Q4 (TC > 5.40 mmol/l, TG > 1.70 mmol/l, and LDL > 3.30 mmol/l) had a higher BMI and BP, BUN, SUA and FPG levels than those in Q1 (TC ≤ 4.20 mmol/l, TG ≤ 0.80 mmol/l, and LDL ≤ 2.30 mmol/l). Q4 also had a higher prevalence of those who were overweight, hypertensive and had obesity, as well as a lower eGFR, than those in Q1.

**Table 2:** Baseline characteristics of subjects by quartiles of TC

TC (mmol/l)	Q1(≤4.20) (***n*** = 1315)	Q2(> 4.20, ≤ 4.80) (***n*** = 1287)	Q3(> 4.80, ≤ 5.40) (***n*** = 1292)	Q4(> 5.40) (***n*** = 1289)	***P*** value
Age (Years)	38(18)	46(22)^*^	50(23)^*#^	55(21)^*#※^	< 0.001
Male (%)	833(63.4)	809(62.9)^*^	800(61.9)^*#^	734(56.9)^*#※^	< 0.001
BMI (kg/m^2^)	22.54 ± 2.95	23.12 ± 2.94^*^	23.48 ± 2.95^*#^	23.84 ± 3.11^*#※^	< 0.001
Overweight (24 ≤ BMI < 28) (%)	340(25.9)	418(32.5)^*^	471(36.5)^*#^	502(38.9)^*#※^	< 0.001
Obesity (BMI ≥ 28) (%)	56(4.3)	64(5.0)^*^	77(6.0)^*#^	114(8.8)^*#※^	< 0.001
SBP (mmHg)	120.27 ± 16.71	124.36 ± 17.67^*^	126.50 ± 18.83^*#^	130.88 ± 19.42^*#※^	< 0.001
DBP (mmHg)	72.52 ± 10.58	74.97 ± 11.25^*^	76.33 ± 11.38^*#^	78.45 ± 11.24^*#※^	< 0.001
TG (mmol/L)	1.08 ± 0.76	1.28 ± 0.85^*^	1.49 ± 0.96^*#^	1.87 ± 1.27^*#※^	< 0.001
HDL-C (mmol/L)	1.37 ± 0.34	1.32 ± 0.30^*^	1.29 ± 0.29^*#^	1.21 ± 0.25^*#※^	< 0.001
LDL-C (mmol/L)	2.03 ± 0.38	2.55 ± 0.34^*^	2.98 ± 0.37^*#^	3.67 ± 0.67^*#※^	< 0.001
BUN (mmol/L)	4.79 ± 1.07	4.98 ± 1.13^*^	5.07 ± 1.19^*#^	5.15 ± 1.13^*#※^	< 0.001
FPG (mmol/L)	5.08 ± 0.65	5.20 ± 0.81^*^	5.26 ± 0.80^*#^	5.41 ± 0.99^*#※^	< 0.001
SCr (μmol/L)	82.26 ± 13.48	82.75 ± 13.91	83.03 ± 13.61^*^	83.13 ± 13.88^*#^	0.371
SUA (mg/dl)	5.44 ± 1.28	5.56 ± 1.32^*^	5.65 ± 1.36^*#^	5.83 ± 1.39^*#※^	< 0.001
Hypertension (%)	131(10.0)	192(14.9)^*^	227(17.6)^*#^	299(23.1)^*#※^	< 0.001
Diabetes (%)	42(3.2)	53(4.0)^*^	41(3.2)^#^	62(4.8)^*#※^	0.089
eGFR (ml/min/1.73m^2^)	90.81 ± 15.67	87.60 ± 16.09^*^	84.84 ± 14.35^*#^	81.56 ± 13.40^*#※^	< 0.001
New incident CKD (%)	27(2.1)	54(4.2)^*^	43(3.3)^*#^	103(8.0)^*#※^	< 0.001

^*^Compared with Q1 group, *P* < 0.05. ^#^Compared with Q2 group, *P* < 0.05. ^※^Compared with Q3 group, *P* < 0.05

**Table 3:** Baseline characteristics of subjects by quartiles of TG

TG (mmol/l)	Q1(≤0.80) (***n*** = 1279)	Q2(> 0.80, ≤ 1.20) (***n*** = 1326)	Q3(> 1.20, ≤ 1.70) (***n*** = 1284)	Q4(> 1.70) (***n*** = 1294)	***P*** value
Age (Years)	39(18)	48(25)^*^	52(23)^*#^	53(20)^*#※^	< 0.001
Male (%)	551(43.1)	764(57.6)^*^	870(67.8)^*#^	991(76.6)^*#※^	< 0.001
BMI (kg/m^2^)	21.53 ± 2.54	22.68 ± 2.76^*^	23.81 ± 2.93^*#^	24.95 ± 2.74^*#※^	< 0.001
Overweight (24 ≤ BMI < 28) (%)	176(13.8)	384(29.0)^*^	504(39.3)^*#^	667(51.5)^*#※^	< 0.001
Obesity (BMI ≥ 28) (%)	21(1.6)	34(2.6)^*^	97(7.6)^*#^	159(12.3)^*#※^	< 0.001
SBP (mmHg)	117.94 ± 16.05	123.20 ± 18.31^*^	127.34 ± 18.35^*#^	133.42 ± 17.97^*#※^	< 0.001
DBP (mmHg)	70.91 ± 10.34	73.53 ± 10.74^*^	76.83 ± 10.75^*#^	80.94 ± 10.88^*#※^	< 0.001
TC (mmol/L)	4.39 ± 0.79	4.71 ± 0.83^*^	4.99 ± 0.92^*#^	5.23 ± 0.93^*#※^	< 0.001
HDL-C (mmol/L)	1.48 ± 0.31	1.36 ± 0.29^*^	1.24 ± 0.26^*#^	1.10 ± 0.22^*#※^	< 0.001
LDL-C (mmol/L)	2.45 ± 0.64	2.79 ± 0.70^*^	2.94 ± 0.79^*#^	3.04 ± 0.76^*#※^	< 0.001
BUN (mmol/L)	4.95 ± 1.20	4.97 ± 1.15	5.01 ± 1.12^*^	5.06 ± 1.09^*#^	0.052
FPG (mmol/L)	5.04 ± 0.56	5.16 ± 0.76^*^	5.26 ± 0.73^*#^	5.49 ± 1.10^*#※^	< 0.001
SCr (μmol/L)	77.74 ± 13.81	81.56 ± 13.46^*^	84.63 ± 12.86^*#^	87.32 ± 12.81^*#※^	< 0.001
SUA (mg/dl)	4.96 ± 1.12	5.36 ± 1.27^*^	5.80 ± 1.25^*#^	6.35 ± 1.32^*#※^	< 0.001
Hypertension (%)	89(7.0)	168(12.7)^*^	253(19.7)^*#^	339(26.2)^*#※^	< 0.001
Diabetes (%)	29(2.3)	39(2.9)^*^	56(4.4)^*#^	74(5.6)^*#※^	< 0.001
eGFR (ml/min/1.73m^2^)	92.36 ± 16.27	86.79 ± 14.98^*^	83.71 ± 13.87^*#^	82.09 ± 13.96^*#※^	< 0.001
New incident CKD (%)	39(3.0)	37(2.8)^*^	54(4.2)^*#^	97(7.5)^*#※^	< 0.001

^*^Compared with Q1 group, *P* < 0.05. ^#^Compared with Q2 group, *P* < 0.05. ^※^Compared with Q3 group, *P* < 0.05

**Table 4:** Baseline characteristics of subjects by quartiles of LDL

LDL (mmol/l)	Q1(≤2.30) (***n*** = 1321)	Q2(> 2.30, ≤ 2.80) (n = 1292)	Q3(> 2.80, ≤ 3.30) (***n*** = 1280)	Q4(> 3.30) (***n*** = 1290)	***P*** value
Age (Years)	40(19)	46(23)^*^	50(23)^*#^	54(18)^*#※^	< 0.001
Male (%)	745(56.4)	781(60.4)^*^	837(65.4)^*#^	813(63.0)^*#※^	< 0.001
BMI (kg/m^2^)	22.29 ± 2.93	23.01 ± 2.98^*^	23.59 ± 2.91^*#^	24.10 ± 2.98^*#※^	< 0.001
Overweight (24 ≤ BMI < 28) (%)	318(24.1)	386(29.9)^*^	487(38.0)^*#^	540(41.9)^*#※^	< 0.001
Obesity (BMI ≥ 28) (%)	49(3.7)	61(4.7)^*^	84(6.6)^*#^	117(9.1)^*#※^	< 0.001
SBP (mmHg)	121.53 ± 18.07	123.60 ± 17.72^*^	126.75 ± 18.62^*#^	130.13 ± 19.13^*#※^	< 0.001
DBP (mmHg)	73.26 ± 11.35	74.38 ± 11.00^*^	76.52 ± 11.34^*#^	78.11 ± 10.95^*#※^	< 0.001
TC (mmol/L)	3.91 ± 0.58	4.51 ± 0.45^*^	5.01 ± 0.42^*#^	5.92 ± 0.71^*#※^	< 0.001
TG (mmol/L)	1.35 ± 1.40	1.35 ± 0.96	1.43 ± 0.80^*#^	1.59 ± 0.78^*#※^	< 0.001
HDL-C (mmol/L)	1.31 ± 0.34	1.30 ± 0.32	1.28 ± 0.29^*^	1.29 ± 0.26^*^	0.065
BUN (mmol/L)	4.81 ± 1.11	4.96 ± 1.14^*^	5.03 ± 1.14^*#^	5.20 ± 1.13^*#※^	< 0.001
FPG (mmol/L)	5.12 ± 0.81	5.17 ± 0.73^*^	5.28 ± 0.85^*#^	5.38 ± 0.89^*#※^	< 0.001
SCr (μmol/L)	80.73 ± 13.83	82.56 ± 13.73^*^	83.82 ± 13.63^*#^	84.11 ± 13.41^*#^	< 0.001
SUA (mg/dl)	5.35 ± 1.33	5.54 ± 1.31^*^	5.69 ± 1.34^*#^	5.90 ± 1.35^*#※^	< 0.001
Hypertension (%)	164(12.4)	190(14.7)^*^	209(16.3)^*#^	286(22.2)^*#※^	< 0.001
Diabetes (%)	44(3.3)	40(3.1)	57(4.5)^*#^	57(4.4)^*#^	0.147
eGFR (ml/min/1.73m^2^)	90.39 ± 16.53	87.05 ± 15.37^*^	85.29 ± 14.53^*#^	82.06 ± 13.37^*#※^	< 0.001
New incident CKD (%)	49(3.7)	49(3.8)	42(3.3)^*#^	87(6.7)^*#※^	< 0.001

^*^Compared with Q1 group, *P* < 0.05. ^#^Compared with Q2 group, *P* < 0.05. ^※^Compared with Q3 group, *P* < 0.05

A comparison of baseline characteristics of participants with and without incident CKD is shown in Table 5. During the 6-year follow-up period, 227 patients (4.4%) developed CKD. Participants with CKD were older and more likely to be obese, and have hyperlipidemia, hypertension or diabetes than patients without CKD.

**Table 5:** Baseline characteristics of participants relative to development of CKD during the 6-year follow-up period

	CKD (***n*** = 227)	Without CKD (***n*** = 4956)	***P*** value
Age (Years)	72(23)	47(22)	< 0.001
Male (%)	139(61.2)	3037(61.3)	0.989
BMI (kg/m^2^)	24.32 ± 2.78	23.18 ± 3.01	< 0.001
SBP (mmHg)	139.80 ± 20.81	124.91 ± 18.79	< 0.001
DBP (mmHg)	80.61 ± 12.02	75.31 ± 11.21	< 0.001
TG (mmol/L)	1.71 ± 1.29	1.42 ± 1.11	0.001
ΔTG	0.25 ± 0.28	0.11 ± 0.16	< 0.001
TC (mmol/L)	5.12 ± 1.02	4.83 ± 0.91	< 0.001
ΔTC (mmol/L)	0.27 ± 0.36	0.15 ± 0.23	< 0.001
HDL-C (mmol/L)	1.31 ± 0.28	1.30 ± 0.31	0.048
LDL (mmol/L)	2.98 ± 0.82	2.82 ± 0.77	< 0.001
ΔLDL (mmol/L)	0.15 ± 0.25	0.08 ± 0.19	< 0.001
BUN (mmol/L)	5.68 ± 1.17	4.96 ± 1.14	< 0.001
FPG (mmol/L)	5.46 ± 1.01	5.24 ± 0.82	< 0.001
SUA (mg/dl)	6.24 ± 1.41	5.59 ± 1.33	< 0.001
Hypertension (%)	103(45.4)	746(15.1)	< 0.001
Diabetes (%)	25(11.0)	173(3.5)	< 0.001
Baseline eGFR (ml/min/1.73m^2^)	68.71 ± 7.62	87.01 ± 15.12	< 0.001
Ending eGFR (ml/min/1.73m^2^)	48.75 ± 8.76	72.38 ± 10.99	< 0.001

ΔTG, ΔTC and ΔLDL-C: ending TG/ TC/ LDL minus baseline TG/ TC/ LDL, respectively

### Incidence of CKD and blood lipid levels

**Fig. 3:**
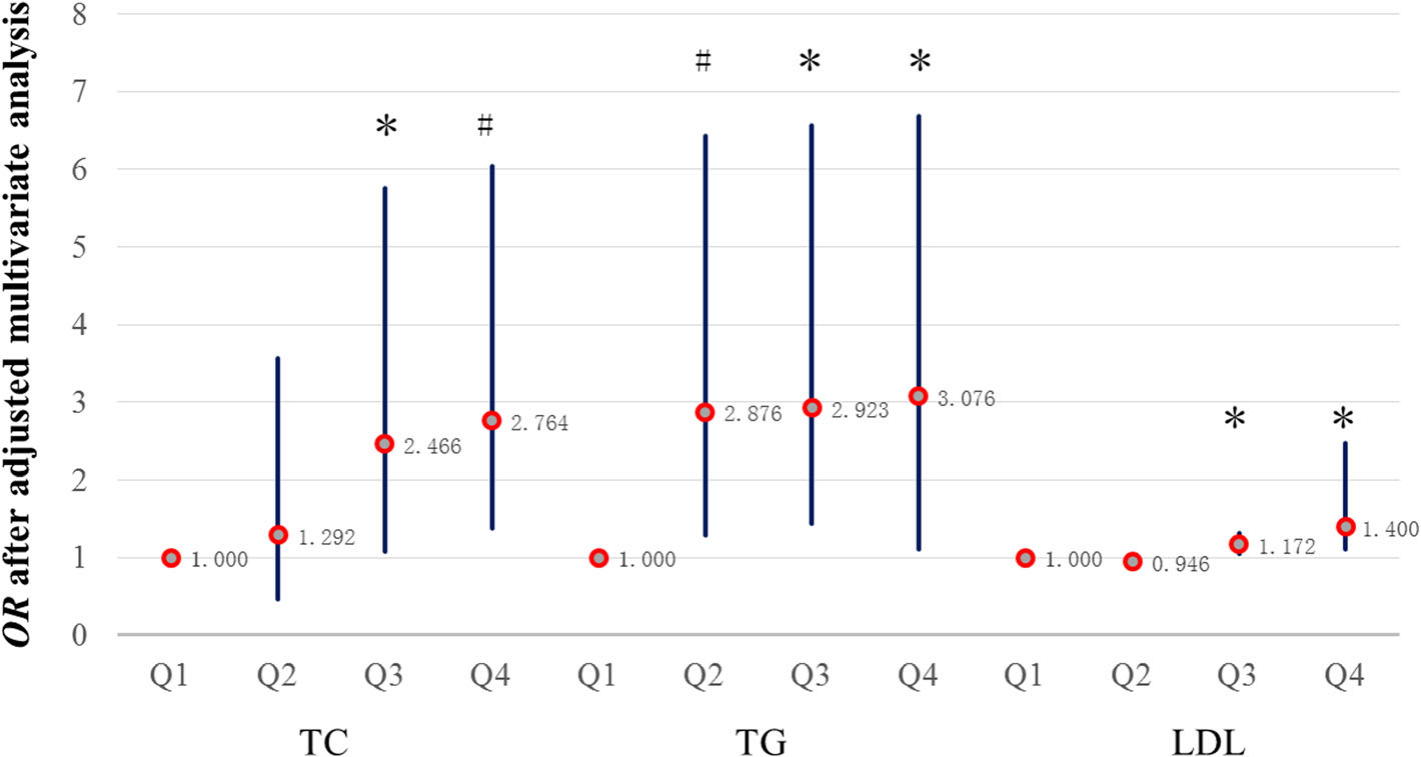
The relationship between blood lipid levels and OR after multivariable-adjusted analysis. A comparison of Q2–4 and Q1 in the same lipid group, respectively. Group TC: Data adjusted for age, gender, BMI, BP, TG, HDL, LDL, BUN, FPG, SCr, SUA, hypertension, diabetes, proteinuria. Group TG: Data adjusted for age, gender, BMI, BP, TC, HDL, LDL, BUN, FPG, SCr, SUA, hypertension, diabetes, proteinuria. Group LDL: Data adjusted for age, gender, BMI, BP, TC, TG, HDL, BUN, FPG, SCr, SUA, hypertension, diabetes, proteinuria. ^*^P < 0.05, ^#^*P* < 0.01

We compared and analyzed the relationship between blood lipid levels and incident CKD (Fig. 3).

In the TC group, with or without adjustment, when TC was greater than 4.76 mmol/l, the likelihood of developing CKD increased commensurate with the increase in TC levels (the OR and 95% CI for the Q3 and Q4 groups were 3.22 [95%CI: 1.57–6.60] and 3.96 [95%CI: 1.97–7.99], respectively, *P* < 0.01), compared with Q1 as a reference. We also obtained the same result after adjusting in the multivariate model, but the OR was reduced (the OR and 95% CI for Q3 was 2.47 [95%CI: 1.075–5.66], *P* < 0.05, and in Q4 it was 2.76 [95%CI: 1.38–6.04], *P* < 0.01) (Fig. 3). This indicates that high TC levels had an impact on renal dysfunction.

Similarly, in the LDL group, development of CKD increased commensurate with the increase in LDL levels (the OR and 95% CI for the Q2, Q3 and Q4 groups were 1.38 [95%CI: 1.08–1.84], 1.52 [95%CI: 1.18–2.03] and 1.88 [95%CI: 1.31–2.69], respectively, *P* < 0.01), compared with Q1 as a reference. However, after adjusting in the multivariable model, only the Q3 and Q4 groups were significantly different (the OR and 95% CI for the Q3 and Q4 groups were 1.17 [95%CI: 1.04–1.32] and 1.40 [95%CI: 1.11–2.48], respectively, *P* < 0.05) (Fig. 3).

In the TG group, development of CKD increased commensurate with the increase in TG levels (the OR and 95% CI for the Q2, Q3 and Q4 groups were 3.025 [95%CI: 1.47–6.21], 3.15 [95%CI: 1.55–6.61] and 3.35 [95%CI: 1.64–6.83], respectively, *P* < 0.01), compared with Q1 as a reference. After we adjusted for baseline SCr, the OR decreased to 2.98 (95%CI: 1.50–6.11) in Q2, 3.11 (95%CI: 1.49–6.325) and 3.24 (95%CI: 1.59–6.62) in the Q4 group, while it significantly decreased to 2.88 (95%CI: 1.29–6.43) in Q2, 2.92 (95%CI: 1.44–6.57) in Q3, and 3.08 (95%CI: 1.11–6.69) in Q4 after adjustment in the multivariate model (Fig. 3).

## Discussion

Before this study, we used the similar data to analyze the relationship between serum uric acid and CKD in the general population, and concluded that time-mean serum uric acid is an independent risk factor for CKD [18]. Dyslipidemia and hyperuricemia are important diseases that affect human health [19]. Therefore, we specifically studied the relationship between dyslipidemia and CKD.

In the current study, we found that TG, and high levels of TC and LDL were associated with the occurrence of CKD, while low TC and LDL levels were not. In particular, the primary findings of our study suggest that TG, and high levels of TC and LDL indicate a risk of progression of renal progression and renal dysfunction, even within the normal range of TG levels. Secondly, the results of this study show that after adjusting for confounding factors, the effect of high blood lipid levels on new incident CKD is greater than low blood lipids, which is consistent with the “lipid nephrotoxicity hypothesis”. This study also showed that there was a statistical difference between blood lipid levels and the incidence of CKD. Participants with the highest quartile of TC and TG in Q4 had a 1–2 times higher risk of CKD than those with the lowest quartile of Q1, and LDL were nearly doubled. Increasing blood lipid levels will accelerate the development of ESRD.

In many studies, the causal role between dyslipidemia and the occurrence and progression of CKD appears to be clear [20–22]. However, the role for the type of blood lipids seems to be conflicting in the occurrence and development of CKD. A study has shown that elevated TC, TG, and LDL are significant risk factors for an eGFR decline in apparently healthy children and adolescents [23]. Recently, Kuma et al. studied the association of dyslipidemia and eGFR reduction in 14,510 healthy people aged 20–60 years and found that elevated LDL-C i.e. exceeding 7.78 mmol/l (140 mg/dL) was a significant risk factor for development of CKD during follow-up over 5 years [24]. In 2015, a follow-up study of 1824 subjects over 5 years found that hypertriglyceridemia was associated with CKD in Japan [25]. Similarly, Tsuruya et al. conducted a prospective cohort study of 117,279 participants, including 47,373 males and 69,422 females, aged 39–74 years. This study showed that hypertriglyceridemia could lead to the progression of CKD [26]. However, Tozawa et al. found that TC and LDL were not independent risk factors for proteinuria [27] and Iseki also found that hypercholesterolemia was not an independent predictor of ESRD [28].

The mechanism of dyslipidemia leading to CKD is still being improved. Current studies have shown that abnormal lipids in blood lead to the accumulation of ectopic lipids, which can be deposited in almost all cell types from mesangial cells to podocytes and proximal tubular epithelial cells [29]. Lipid-induced mitochondrial damage may also be more lethal to proximal tubule cells [30]. High cholesterol causes macrophage infiltration and foam cell formation in the kidney. The accumulation of triglycerides and lipid metabolism breakdown products in the blood of CKD patients has a strong atherosclerosis and pro-inflammatory effect on the vascular system in the renal parenchyma [31]. In addition, CD36 is highly expressed in proximal and distal renal tubular epithelial cells, podocytes, mesangial cells, microvascular endothelial cells and interstitial macrophages, which can mediate the uptake of oxidized LDL (ox-LDL) [32], and also can be combined with various circulating ligands to promote the development of kidney inflammation, oxidative stress and fibrosis [33, 34]. Recently, it has been demonstrated that the overexpression of CD36 transgene in mouse kidney induces the accumulation of lipid in the kidney [35]. CD36-mediated signaling pathway causes proteinuria-induced injury of tubulointerstitial [36].

We studied the Zhejiang population in China this time. Zhejiang has a higher standard of living than other parts of China, and residents have high levels of fat in their diets, leading to more cases of hyperlipidemia. Moreover, the medical level in Zhejiang is relatively high. Residents also pay more attention to their health status. Therefore, there are more elderly people. Thus, this study is mainly aimed at Zhejiang, China. And if we have the opportunity, we will also carry out large-scale national research to further explain the situation of the China, and also enrich the hypothesis of lipid nephrotoxicity.

However, it is important to acknowledge that our research also has certain limitations. Participants who participated in the annual physical examination were very concerned about their own health. It was likely that some of the incident CKD patients did not participate in the next annual physical examination but may have been followed-up in the outpatient clinic. Moreover, we cannot fully include all factors that affect blood lipids and renal function. In short, large-scale research is imperative.

## Conclusion

In conclusion, we found that TG, and high levels of TC and LDL are associated with decreased renal function and an increased likelihood of incident CKD development in the general population. This demonstrated that controlling elevated TG, TC, and LDL might contribute to reduced propensity of renal dysfunction. Monitoring and management of blood lipids on a regular basis, especially TG, should be considered.

## Data Availability

All data supporting the study are presented in the manuscript and available on a request to the corresponding authors of this manuscript, Qiang He.

## Abbreviations

eGFR: Estimated glomerular filtration rate
CKD: Chronic kidney disease
ESRD: End-stage renal disease
CVD: Cardiovascular disease
TG: Triglycerides
TC: Total cholesterol
LDL: Low-density lipoprotein
BMI: Body mass index
SBP/DBP: Systolic and diastolic blood pressure
BP: Blood pressure
FPG: Fasting blood glucose
HDL: High-density lipoprotein
SUA: Uric acid
MDRD: Modification of Diet in Renal Disease
KDIGO: Kidney Disease: Improving Global Outcomes
